# Tone language experience enhances dimension-selective attention and subcortical encoding but not cortical entrainment to pitch

**DOI:** 10.1162/imag_a_00297

**Published:** 2024-10-01

**Authors:** Magdalena Kachlicka, Ashley E. Symons, Kazuya Saito, Frederic Dick, Adam T. Tierney

**Affiliations:** Department of Psychological Sciences, Birkbeck, University of London, London, United Kingdom; Department of Psychology, Royal Holloway, University of London, London, United Kingdom; Institute of Education, University College London, London, United Kingdom; Division of Psychology and Language Sciences, University College London, London, United Kingdom

**Keywords:** cue weighting, attention, salience, second language

## Abstract

What factors determine the importance placed on different sources of evidence during speech and music perception? Attention-to-dimension theories suggest that, through prolonged exposure to their first language (L1), listeners become biased to attend to acoustic dimensions especially informative in that language. Given that selective attention can modulate cortical tracking of sounds, attention-to-dimension accounts predict that tone language speakers would show greater cortical tracking of pitch in L2 speech, even when it is not task-relevant, as well as an enhanced ability to attend to pitch in both speech and music. Here, we test these hypotheses by examining neural sound encoding, dimension-selective attention, and cue-weighting strategies in 54 native English and 60 Mandarin Chinese speakers. Our results show that Mandarin speakers, compared to native English speakers, are better at attending to pitch and worse at attending to duration in verbal and non-verbal stimuli; moreover, they place more importance on pitch and less on duration during speech and music categorization. The effects of language background were moderated by musical experience, however, with Mandarin-speaking musicians better able to attend to duration and using duration more as a cue to phrase boundary perception. There was no effect of L1 on cortical tracking of acoustic dimensions. Nevertheless, the frequency-following response to stimulus pitch was enhanced in Mandarin speakers, suggesting that speaking a tone language can boost processing of early pitch encoding. These findings suggest that tone language experience does not increase the tendency for pitch to capture attention, regardless of task; instead, tone language speakers may benefit from an enhanced ability to direct attention to pitch when it is task-relevant, without affecting pitch salience.

## 1 Introduction

Prior research suggests that first language (L1) background shapes perceptual strategies. Lifelong exposure to L1-specific distributional information tunes the auditory system to acoustic dimensions that carry relevant information (Holt & Lotto, 2006), making individuals experts in weighting their importance according to how reliably they predict category membership (Francis et al., 2000;Toscano & McMurray, 2010). These dimensions are acoustic or perceptual qualities, such as pitch, duration, or amplitude, that vary across a range of values. Different values along these dimensions can serve as cues for disambiguating alternative interpretations of perceptual objects or classes. One striking difference in cue use emerges between tonal and non-tonal languages. While tonal languages use contrastive fundamental frequency variation to mark lexical tones, pitch contour plays a more secondary role in non-tonal languages (Francis et al., 2008;Y.-C. Hao, 2018), typically conveying prosody (linguistic focus,Breen et al., 2010; statements and questions,Bartels, 1999) and emotional states (e.g.,Rodero, 2011) and providing a minor cue to stop-consonant voicing (Haggard et al., 1970); in each of these cases, pitch is accompanied by cues in other dimensions such as relative duration and amplitude. Due to these discrepancies in the relative importance of acoustic dimensions across languages, an optimal L1 listening strategy will not always be as effective for learning a second language (L2).

One possible mechanism underlying the formation of perceptual strategies is that expertise in perceiving acoustic variations along L1-relevant dimensions enhances their relative salience, or tendency to capture attention regardless of task, leading to upweighting of that dimension during perception (Francis & Nusbaum, 2002;Gordon et al., 1993;Holt et al., 2018). Preliminary support for these attention-to-dimension models comes from recent work on Mandarin Chinese speakers, who place more importance on pitch contour and less on other acoustic information while listening to English stress (Wang, 2008;Yu & Andruski, 2010;Y. Zhang & Francis, 2010) and phrase boundaries (Jasmin, Sun, & Tierney, 2021;Zhang, 2012), and overuse pitch contour in speech production (Nguyễn et al., 2008;Y. Zhang et al., 2008). Importantly, these shifts in perceptual strategies extend to music categorization tasks; moreover, Mandarin speakers have difficulty ignoring pitch contour and attending to other dimensions in speech, even when explicitly instructed to do so (Jasmin, Sun, & Tierney, 2021), suggesting that tone language experience might be linked to increased pitch contour salience.

Musical training might also contribute to differences in dimension weighting strategies (OPERA hypothesis;Patel, 2014;Patel & Iversen, 2014) since it involves learning to selectively attend to certain single acoustic dimensions which convey particularly important information in music. In speech, information is generally conveyed across multiple dimensions simultaneously (Winter, 2014). This is the case for certain structural features in music as well, such as beat strength and musical phrase boundaries, which are conveyed by pitch and duration cues (Ellis & Jones, 2009;Tierney et al., 2011). However, other music perception tasks require very precise tracking of information from a single acoustic dimension, with no available redundancy from other dimensions. For example, perception of one semitone pitch differences is vital for tracking harmony (Trainor & Trehub, 1994), and musicians can correct for synchronization timing errors of as little as 1.5 ms (Madison & Merker, 2004). The necessity of directing attention to single acoustic dimensions during music perception and performance may lead to a link between musical training and enhanced dimension-selective attention. Supporting this idea,Symons and Tierney (2023)demonstrated that musical experience is linked to enhanced attention to task-relevant dimensions and increased use of the most useful primary dimension for a given suprasegmental categorization task—pitch for word emphasis perception, but duration for phrase boundary perception. These results suggest that, unlike experience speaking a tone language, musical experience does not increase the salience of a particular dimension, but instead improves the ability to flexibly attend to the most useful dimension for a given task, leading musicians to adopt perceptual strategies in which they use one cue to the relative exclusion of all others.

### 1.1 Present study

The primary goal of this study was to test the hypothesis that tone language experience modulates perceptual strategies by changing the salience of acoustic dimensions. We test this hypothesis in the context of pitch and duration—pitch is a highly relevant dimension in Mandarin Chinese but has secondary importance in English; duration was chosen as a dimension orthogonal to pitch in speech. We compared neural encoding of those dimensions, participants’ selective attention to pitch and duration, and cue-weighting during prosody and music categorization. Departing from traditional methods of measuring salience using behavioral ratings (Kaya & Elhilali, 2014), we measured dimensional salience with an EEG frequency tagging paradigm, in which different dimensions within a single sound stream changed at different rates. The frequency tagging paradigm was originally developed to quantify attentional modulation of neural responses to visual stimuli by measuring potentials elicited through the presentation of stimuli with distinctive flicker frequencies (Toffanin et al., 2009). More recently, tagging stimuli at different presentation rates has been adapted for neural tracking of changes within sounds (i.e., acoustic dimensions can be targets of attention) as well as tracking of competing speech streams (Bharadwaj et al., 2014), linguistic structures (Ding et al., 2016), and neural entrainment to beat and meter (Nozaradan et al., 2011). Recent research using this paradigm has shown that dimensional salience and dimension-selective attention modulate cortical tracking specifically at the rate tagged to that dimension (duration and intensity,Costa-Faidella et al., 2017; pitch and spectral peak,Symons et al., 2021). Prior research has found that tone language speakers have enhanced early encoding of pitch, as measured using frequency-following responses (FFRs;Krishnan et al., 2010); however, the effects of tone language experience on cortical tracking of pitch in speech remain unclear. We predicted that Mandarin speakers would exhibit not only stronger early encoding of pitch in the FFR, but also stronger cortical tracking of pitch across both verbal and non-verbal stimuli and weaker tracking of duration. We further predicted that Mandarin speakers would demonstrate an enhanced ability to attend to pitch but would struggle to ignore pitch and attend to duration, in both verbal and non-verbal sounds. Finally, we predicted that Mandarin speakers would demonstrate increased pitch weighting across multiple speech perception tasks (categorization of stress, word emphasis, and phrase boundaries) as well as during musical beat perception.

On the other hand, given that prior research found that musical training was linked to an enhanced ability to focus on a single*task-relevant*dimension rather than a global up-weighting of a single dimension (Symons & Tierney, 2023), we did not predict that musical training would relate to changes in dimensional salience. Instead, given prior findings of enhanced attentional skills in musicians (Micheyl et al., 2006), we predicted that musicians would demonstrate an enhanced ability to attend to auditory dimensions in general, as well as a tendency to highly weight the primary dimension that serves as a cue to a given categorization task, down-weighting secondary sources of information. Moreover, investigating both L1 background and musical training enabled us to test the interaction between these two types of experience and their role in shaping listening strategies.

## 2 Methods

### 2.1 Participants

The group of English native speakers comprised students recruited from the SONA platform for participant recruitment (Sona Systems,https://www.sona-systems.com/) and professional musicians recruited from music job boards. Mandarin speakers were students recruited from the SONA platform and social media community groups (Facebook and WeChat). A total of 61 English speakers and 75 Mandarin speakers completed the study; however, only the data from 54 English and 60 Mandarin speakers were included in the analyses (The EEG and dimension-selective attention data from the English speakers were previously reported as Experiment 1 inSymons et al., 2023). The dataset comprises the neural and behavioral data from all participants (i.e., all participants completed all the tasks). Participants who in the categorization tasks showed either a significant negative correlation between either stimulus dimension and categorization responses (p < .05) or no significant relationship between either stimulus dimension or categorization responses (patterns suggestive of misunderstanding task instructions) were flagged for removal. Five Mandarin speakers were excluded based on poor performance in the dimension-selective attention tasks (< 75% correct responses in the single dimension training blocks after three attempts), and 10 were excluded based on their responses in categorization tasks. Six English speakers were excluded based on their responses in categorization tasks and one due to technical issues that prevented the researcher from recording their EEG data. Most of the effects reported in this manuscript hold when analyses were conducted on the full set of participants who completed the study. The only exception is the main effect of L1 on high-frequency EEG noise, which did not reach significance (p = .071).

Most English-native-speaking participants (aged 18–38; M = 23.94, SD = 5.62; 37 females, 17 males) were raised speaking only English. Only 6 of them indicated speaking another language since birth (one Farsi, one Portuguese, one Russian, and three Bengali speakers), whereas 28 studied at least one other language starting from teenage years to early adulthood (e.g., Spanish, German, French, Portuguese, Hebrew, Russian, Italian). None of the participants had previous experience with tonal languages. Following the criteria described byZhang, Susino, et al. (2020), we considered as musicians only the participants who reported more than 6 years of systematic musical training (N = 29). Most English-native-speaking musicians reported playing more than one instrument (only six played one instrument, and three were professional singers). Most of them played either guitar or piano (N = 15 for each instrument), and the rest played a variety of other instruments (bass, clarinet, drums, violin, flute, trumpet, harp, oboe, recorder, cello, horn, bassoon, or accordion). Of the non-musicians, nine participants reported practicing music in their childhood, but stated that they were no longer able to play any instrument and the remaining participants had no musical training.

Mandarin speakers (aged 18–31; M = 22.62, SD = 3.27; 53 female, 6 male, 1 non-conforming) all spoke English as a second language but were not raised bilingually – they learned English at school and reported only 1 to 17 months (M = 7.41, SD = 3.21) of residence in English-speaking countries. While much L2 learning could happen within the first few months of immersion, the link between the amount of immersion and L2 speech learning is subject to a great deal of individual variation (Munro & Derwing, 2008). Seven participants reported speaking an additional language (one Russian, one French, one German, two Japanese, and two Korean). Twenty-nine Mandarin-speaking participants reported more than 6 years of musical training, compared to non-musicians who had little to no music experience (eight participants reported practicing music in the past but stated they are currently unable to play any instruments). Most participants with musical training reported playing piano (N = 15); the remaining participants played various instruments such as violin, pipe, flute, guitar, bass, or clarinet and were trained in singing, and five participants were trained to play traditional Chinese instruments. Ten participants reported playing more than one instrument.

### 2.2 Behavioral measures

#### 2.2.1 Dimension-selective attention task

##### 2.2.1.1 Task

This task was designed to measure participants’ ability to pay attention to changes along one acoustic dimension while ignoring changes in another dimension. Participants listened to sequences of verbal (speech) and non-verbal (tones) sounds changing in pitch and duration at two different rates. At the beginning of each block, they were asked to pay attention to changes in one of the acoustic dimensions. Once the stimulus had finished playing, text appeared on the screen asking participants whether they heard a repetition within the attended dimension. Participants responded by clicking the “Yes” or “No” button on the screen. Feedback was provided on each trial. Participants received the next set of instructions between blocks and could take a break.

Prior to the task, participants listened to examples of different pitch and duration levels and sequences where only a single dimension was changing. Participants then completed a short training task with these sequences. The training task was blocked by attention conditions but with the rate of the attended dimension randomized. At the start of each block, participants were informed which dimension to attend to and the rate at which that dimension was expected to vary. Participants received eight trials per attention condition (four per rate). Participants were required to answer at least six out of eight trials (75%) correctly on each training module to move on to the next task. If participants failed to reach the performance threshold, they could repeat the training for that dimension up to three times, and they were not allowed to continue to the next stage of the study if they failed to do so.

Trials in the main task were identical to the training task except that both dimensions were changing. For each stimulus type (speech and tones), 1 block of each of 4 conditions was presented in random order (2 attention conditions x 2 rates of change). At the start of each block, participants were told which dimension to attend to and the rate at which that dimension was expected to vary. Participants’ responses were recorded, and the proportion of correct responses (collapsed across dimension change rate) for each dimension was computed as the dependent variables.

##### 2.2.1.2 Stimuli

The base stimuli were eight unique tokens, four speech sounds and four tones, varying along fundamental frequency (F0) and duration. Verbal tokens were generated by extracting vowels from speech excerpts, and non-verbal tokens were acoustically matched synthesized tones. The speech stimuli were extracted from the phrase "Tom likes barbecue chicken" taken from the Multidimensional Battery of Prosody Perception (MBOPP;Jasmin, Dick, & Tierney, 2021). We used two versions of this phrase, with and without emphasis placed on the word "barbecue” and extracted the first vowel /a/ from both versions to capture clearly audible natural within-vowel pitch and duration variations. To create pitch-varying stimuli, we morphed the emphasized and non-emphasized vowels along the F0 dimension using STRAIGHT (Kawahara & Irino, 2005) by extracting the F0 from voiced parts of the recordings and analyzing periodic aspects and filter characteristics of the signal. Finally, corresponding salient portions of the recordings (i.e., anchor points) were manually marked, and 100 morphed samples were generated, representing a smooth transition of F0 values from the emphasized to non-emphasized vowels. Duration and other acoustic parameters were kept constant. We selected two samples that differed from each other by approximately 2 semitones (Level 1 = 110.88 Hz and Level 56 = 124.40 Hz; difference = 2.03 semitones) to make the differences easily perceivable by all participants. Then, we used Praat (Boersma & Weenink, 2023) to morph the duration of the vowel to 70.58 and 175.83 ms (difference = 105 ms) and created a 2 (pitch) x 2 (duration) stimulus grid using the selected stimuli. These F0/duration values were selected to balance the relative salience of the pitch/duration differences, as judged by the authors. The non-verbal stimuli were complex tones with four harmonics with acoustic properties matching the speech stimuli. The tones varied along two dimensions: F0 (110.88 and 124.72 Hz) and duration (70 and 175 ms). All stimuli were ramped with 10-ms on/off cosine ramps.

**Fig. 1.**
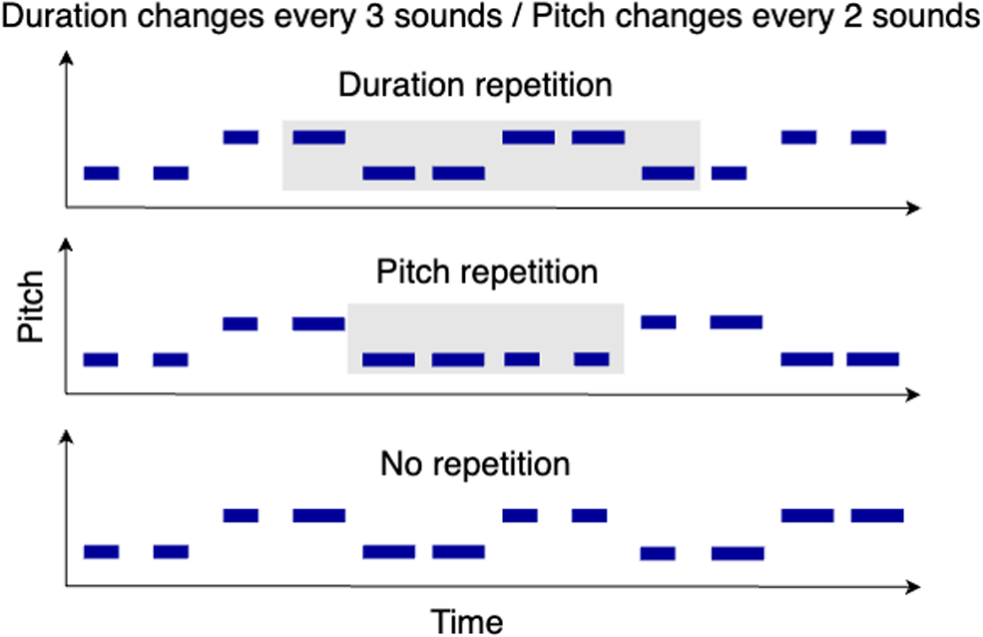
Schematic of example sequences from the dimension-selective attention task. In all examples, duration changes every three sounds and pitch every two sounds. (Top) Example sequence with duration repetition highlighted in grey. (Middle) Example sequence with pitch repetition highlighted in grey. (Bottom) Example sequence without repetition.

Stimuli were concatenated to form sequences of sounds with a presentation rate of 2 Hz, with pitch and duration changing at different rates (every three sounds = 0.67 Hz and every two sounds = 1 Hz). Repetitions, or instances where the dimension did not change at the expected time, were inserted into half of the sequences for each dimension (Fig. 1). This resulted in four trial types: pitch repetition only, duration repetition only, repetitions in both dimensions, and no repetitions in either dimension. The stimuli in each domain and attention condition were identical, varying only in the focus of attention. From each stimulus set (speech and tones), 64 stimuli (32 varying in pitch at 1 Hz and duration at 0.67 Hz and 32 varying in duration at 1 Hz and pitch at 0.67 Hz) were randomly selected and assigned to either attend pitch or attend duration conditions (32 trials per condition). The stimuli were assigned to the opposite attention conditions in two versions of the task to counterbalance items across subjects.

#### 2.2.2 Prosodic cue weighting tasks

##### 2.2.2.1 Task

Participants completed four cue weighting tasks representing three prosodic features (phrase boundary, linguistic focus, lexical stress) and musical beats. In all four categorization tasks, participants were presented with stimuli that varied orthogonally in the extent to which F0 and duration were indicators of one of the two possible categories. After listening to each stimulus, participants were asked to categorize the stimuli as belonging to one of two categories: phrase with early or late closure ("If Barbara gives up, the ship" vs. "If Barbara gives up the ship"), emphasis on the first or second word ("STUDY music" vs. "study MUSIC"), lexical stress on the first versus second syllable ("COM-pound" vs. "com-POUND"), and musical beats occurring either every two or three notes ("strong—weak" vs. "strong—weak—weak" patterns). They were provided two written alternatives, and they indicated their choice by pressing an appropriate button on the screen. Before the main task, participants listened to examples of each recording with unaltered pitch and duration and two practice trials with written feedback. The main tasks were identical to the practice except that feedback was no longer provided and all 16 stimuli were presented in random order. There were 10 blocks of each categorization task, which were interleaved in the following order: musical beats, linguistic focus, lexical stress, and phrase boundary. Practice trials were included on the first block of each task but not thereafter. Participants received progress updates after completing one block of each task.

##### 2.2.2.2 Stimuli

Linguistic focus and phrase boundary stimuli were taken from the MBOPP battery (Jasmin, Dick, & Tierney, 2021). Additionally, lexical (syllable) stress stimuli were recorded to complement this dataset, so that all the included sentences captured contrasts across three prosody features. The speech tokens were created by recording the voice of a native Southern British English speaker reading pairs of contrastive phrases (early vs. late linguistic focus: “Dave likes to STUDY music” vs. “Dave likes to study MUSIC”; early vs. late phrase boundary: “If Barbara gives up, the ship will be plundered” vs. “If Barbara gives up the ship, it will be plundered” and first vs. second syllable stress: “COMpound” vs. “comPOUND” embedded within carrier sentences). Identical portions of the recordings (i.e., "study music", "If Barbara gives up the ship", and "compound") were then extracted, and the two versions of the same phrase that differed in the location of the prosodic contrast were morphed together using the MATLAB toolbox STRAIGHT (Jasmin et al., 2020;Kawahara & Irino, 2005) by adjusting the values of F0 and durational morphing rates orthogonally in four steps (i.e., 0%, 33%, 67%, and 100%) to create the stimuli.

The morphing procedure included two steps. First, STRAIGHT generated a “similarity matrix” calculated based on the Mel-frequency cepstral coefficients which displays the similarity between the two recordings across different time points. We then time-aligned the two recordings by visually inspecting their similarity matrix and manually marking anchor points representing corresponding events in each recording (e.g., word and syllable onsets). STRAIGHT then used dynamic time warping to map the two recordings onto one another based on those anchor points, with the constraint that the selected anchor points must align for the remaining frames to be aligned accurately. Next, the time-aligned files with anchor points were used for computing the amount of scaling required to generate the interpolated features and synthesizing the intermediate versions of the sentences which differed in F0 and time, dimensions selected from the list of available options offered by STRAIGHT; all other options were set to be halfway between the two recordings. The researcher listened to the resulting morphed samples, and if the quality was not satisfactory (e.g., there were audible distortions in the created samples), the procedure was repeated until the resulting morphs sounded natural. Scripts used for generating all the stimuli are available at:https://osf.io/ajgrn/.

Musical beats stimuli were sequences of six four-harmonic complex tones (equal amplitude across harmonics, 15-ms on/off cosine ramps) repeated three times. Pitch and duration varied across four levels, indicating either a three-note grouping (“strong—weak—weak” pattern, waltz time) or a two-note grouping (“strong—weak” pattern, march time). The strength of these groupings was determined by the increased pitch or duration of the first tone relative to the other tones of the two- or three-note grouping. The four pitch levels were [C#-A-A-C#-A-A] (representing pitch values that strongly indicated groups of three), [B-A-A-B-A-A], [B-A-B-A-B-A], and [C#-A-C#-A-C#-A] that strongly indicated groups of two, where A equals 440 Hz, B 493.9 Hz, and C# 554.4 Hz. Similarly, the manipulated duration levels varied from [200 50 50 200 50 50 ms] which strongly indicated groups of three, through [100 50 50 100 50 50 ms] and [100 50 100 50 100 50 ms], to [200 50 200 50 200 50 ms] that strongly indicated groups of two.

Stimuli sampled a 4-by-4 acoustic space across duration and F0 so that the acoustic properties of stimuli cued the appropriate categories to four different degrees: 0%, 33%, 67%, and 100%, where 0% values indicate that the F0 or duration values came from Token A recording, 100% means that F0 and duration were identical to the Token B recording, and intermediate values reflect F0 and duration patterns linearly interpolated between the two original recordings. Unlike earlier studies (5 x 5 grid,Jasmin, Sun, & Tierney, 2021; 7 x 7 grid,Jasmin et al., 2023), we do not include the mid-value of ambiguous 50% samples to reduce the time needed to complete the task, necessitated by the overall length of the experiment.

### 2.3 Neural measures

#### 2.3.1 Frequency tagging paradigm

To establish which of the presented dimensions (pitch vs. duration) was more salient to participants while listening to speech and tone sequences, changes in each dimension were tagged to different presentation rates (2.5 or 1.67 Hz), with rate-to-dimension assignment counterbalanced across blocks. Stronger cortical tracking at any given frequency represents the salience of a given dimension changing at that rate (Symons et al., 2021). Additionally, we assessed subcortical pitch encoding across stimuli.

##### 2.3.1.1 Behavioral task

Participants were asked to listen to speech and tone sequences changing in pitch and duration at different rates and respond with keyboard presses to occasional quiet sounds. The purpose of the behavioral task was to keep participants engaged in listening to the stimuli throughout the session, but without directing their attention to pitch or duration.

Before the main task, participants completed a short practice run to familiarize themselves with the task before entering the EEG recording booth. They listened to sequences of speech and tones for about a minute each and continued until they reached at least five out of six correct responses without making too many errors to move to the main task. For the practice, feedback was displayed on the screen, indicating the number of correct and incorrect responses and missed targets. Most participants completed the practice upon their first attempt, and the remaining participants were asked to repeat the practice block. The main task was identical to the practice but with longer sequences and no visual feedback. Behavioral performance was measured to ensure that participants stayed focused throughout the task. There were four blocks, each containing four 2-minute sequences of sounds.

Behavioral data was computed by calculating the proportion of hits and false alarms and converting them to d-prime, using the loglinear approach to prevent infinite scores (Hautus, 1995). Hits were responses within 1.25 seconds following an oddball, while false alarms were responses outside that time frame divided by the total number of non-oddball tones. Behavioral performance was comparable in both conditions: the median d-prime for speech was 3.87, while the median d-prime for tones was 3.67.

##### 2.3.1.2 Stimuli

The base stimuli used for the dimensional salience task were the /a/ vowel with pitch at either 110.88 or 124.4 Hz and short versus long duration (70.58 or 175.83 ms) and acoustically matched synthesized tones (110.88 or 124.72 Hz and 70 or 175 ms). These were the same base stimuli as those used in the dimension-selective attention task. Using the 2 (pitch) x 2 (duration) stimulus grids for each domain (speech and tones), we created 5-Hz sequences (i.e., sound played every 200 ms; 96 seconds in duration) in which pitch and duration changed at fixed rates (every two sounds, 2.5 Hz, or every three sounds, 1.67 Hz). The stimuli consistently varied at these rates apart from 20 repetitions which were inserted into each sequence. These repetitions were inserted to prevent the stimuli from becoming overly predictable but were not task-relevant. For each sequence, the amplitude of 3-5 randomly selected stimuli (32 in total) was decreased by 25% (-12.04 dB) to create amplitude oddballs. Oddball timing was randomized in each sequence, with the exception that oddballs could not occur in the first or last 4.8 seconds (four epochs) of the sequence and could not occur within 4.8 seconds of another oddball. The same sequences were presented to all participants, but with the order counterbalanced across participants. Stimuli were presented diotically at max 80 dB SPL at a sampling rate of 44,100 Hz using PsychoPy3 (v 3.2.3) via 3M E-A-RTONE 3A insert earphones. Stimuli were presented in alternating polarity (half of the stimuli were inverted) so that we could analyze the envelope-following response, in which the representation of lower harmonics is emphasized (Aiken & Picton, 2008).

#### 2.3.2 EEG data acquisition

EEG data were recorded from 32 Ag-Cl active electrodes using a Biosemi™ ActiveTwo system with the 10/20 electrode montage. Data were recorded at a sampling rate of 16,384 Hz and digitized with a 24-bit resolution. Two external reference electrodes were placed on both earlobes for off-line re-referencing. Impedance was kept below 20 kΩ throughout the testing session. All EEG data processing and analysis were carried out in MATLAB (MathWorks, Inc) using the FieldTrip M/EEG analysis toolbox (Oostenveld et al., 2011) in combination with in-house scripts.

#### 2.3.3 Intertrial phase coherence (ITPC)

The data were down sampled to 512 Hz and re-referenced to the average of the earlobe reference electrodes. Down-sampling was performed with the decimate function from the MATLAB Signal Processing Toolbox which uses a low-pass Chebyshev Time I infinite impulse response anti-aliasing prefilter of order 8 (cut-off frequency of 0.025 Hz and passband ripple of 0.05 dB). A low-pass zero-phase sixth-order Butterworth filter with a cutoff of 30 Hz was applied. A high-pass fourth-order zero-phase Butterworth filter with a cut-off of 0.5 Hz was then applied. Data were then divided into non-overlapping 1.2-second epochs. Independent component analysis (ICA) was conducted to correct for eye blinks and horizontal eye movements. Components corresponding to eye blinks and movements were identified and removed based on visual inspection of the time courses and topographies. Any remaining artefacts exceeding +/- 100 μV were rejected. The mean number of remaining epochs did not differ significantly across participant groups (M_Mandarin_= 303.77, SD = 5.80, M_English_= 303.20, SD = 6.50, t(454) = .99, p = .32).

A Hanning-windowed fast Fourier transform was applied to each 1.2-second epoch. The complex vector at each frequency was converted to a unit vector and averaged across trials. The length of the average vector was computed to calculate inter-trial phase coherence (ITPC), which ranges from 0 (no phase consistency) to 1 (perfect phase consistency). The degree of ITPC at the frequency tagged to a given dimension provides indices of dimensional salience (i.e., cortical tracking of acoustic dimensions). Prior to data analysis, we extracted data from the 9 channels with the maximum ITPC when averaged across the two rates of dimensional change (1.67 and 2.5 Hz) and all participants (N = 114). The number of channels to include (i.e., 9) was decided prior to analysis following the standard pre-processing procedures (e.g.,Symons et al., 2021). This resulted in a cluster of frontocentral channels (AF4, F3, Fz, F4, FC1, FC2, FC5, Cz, C3) across which the data were averaged.

#### 2.3.4 Frequency-following response (FFR)

In addition, we analyzed the frequency-following response to assess pitch encoding in the early auditory system. Prior to analysis, we selected data from the central electrode (Cz) and two reference earlobe electrodes from the multi-channel EEG recordings. The data were bandpass filtered with 70 Hz high-pass and 3000 Hz low-pass Butterworth filters. To maximize the number of trials, the data were collapsed across presentation rates and stimulus durations. Moreover, we used all the artefact-free epochs. Such a procedure led to various numbers of trials across participants. However, the mean number of remaining epochs did not differ significantly across participant groups (M_Mandarin_= 7203.33, SD = 536.28, M_English_= 7241.63, SD = 280.88, t(226) = -.66, p = .51). To further maximize the number of epochs for analysis, the data were divided into multiple epochs per stimulus. Specifically, we extracted multiple epochs per stimulus, with non-overlapping windows, each containing three cycles of the F0. Only the FFRs to the speech and tones stimuli with the lower pitch (110.88 Hz) were analyzed, because this stimulus featured a relatively flat pitch contour; the speech stimuli with the higher pitch (124.72 Hz) featured a changing pitch contour, which prevented us from collapsing across F0 cycles. As a result, each epoch was 27 ms long (since a single cycle of a 110.88 Hz F0 lasts 9 ms). Epochs with amplitude above 35 μV were removed. Finally, an equal number of artefact-free epochs taken from responses to each stimulus polarity were selected for analysis.

Inter-trial phase locking was used to measure the precision of neural encoding across trials on a frequency-by-frequency basis (seesection 2.3.3. above for details). ITPC was calculated across trials for frequencies between 200 and 250 Hz; this captured the first harmonic of the fundamental frequency of the stimulus, which was 225 Hz. Our reason for analyzing the first harmonic was that this was the point at which the response was largest, potentially giving us sufficient signal-to-noise for a robust analysis. In addition, we calculated non-phase-locked amplitude as a measure of neural noise (Cohen, 2014). First, the average ERP across all epochs was computed. Next, this average was subtracted from each epoch. The spectral amplitude for each epoch was then measured using an FFT, and the resulting amplitude spectra were averaged across trials. Amplitude between 100 and 500 Hz was extracted as a measure of neural noise.

### 2.4 General procedure

Participants who responded to the study adverts were invited to a short telephone or video call to ensure that they met all the study criteria. Each interview was scheduled individually and during the call, the researcher asked a list of questions about participants’ basic demographics, language, and musical background, explained the experimental procedure and task instructions, and answered participants’ questions. Next, informed consent was obtained from eligible participants, and they received links to online tasks to complete via the Gorilla platform (Anwyl-Irvine et al., 2020). After completing the online tasks, participants were invited to the lab at Birkbeck, University of London for the EEG testing. All procedures were approved by the Ethics Committee for the Department of Psychological Sciences at Birkbeck. All participants were reimbursed for their time in cash (at £10 per hour) or its equivalent in course credits.

### 2.5 Statistical analyses

All statistical analyses were conducted in R. For analysis of cue weighting data, package lmer4 was used for mixed-effects logistic regression models (Bates et al., 2015) quantifying listeners’ use of acoustic cues across categorization tasks. The trial-by-trial responses reflecting categorical decisions (represented as 0 or 1) were used as the dependent variable. The categorical variables representing participants’ L1 background (English, Mandarin) and musical training (non-musicians with less than 6 years of musical training and not currently practicing, musicians with >= 6 years of training) were coded with a scaled sum contrast with the first variable level coded as -0.5 and the second as 0.5. The continuous predictors pitch level (1-4) and duration level (1-4) were standardized by centering and dividing by 2 standard deviations using the rescale function from the arm R package (Gelman et al., 2022). The resulting beta coefficients from the model represent the change in log odds given an increase of one standard deviation of that variable. Participants’ unique IDs were included as a random intercept. Inclusion of random slopes for pitch level and duration level and their interaction resulted in overfitting, so the simpler models without random slopes were selected across categorization tasks. We based our model evaluation on automated warnings from lme4 package that flag instances of “singular fit” in overparametrized models (Bates et al., 2018). Across all models, we only removed terms required to allow for a non-singular fit (as recommended byBarr et al. (2013)).

The glmmTMB function (Brooks et al., 2017) was used for mixed-effects regression models with beta distribution (parameterization ofFerrari & Cribari-Neto, 2004and betareg package;Cribari-Neto & Zeileis, 2010). Using linear models for continuous outcomes bound by 0-1 intervals might result in spurious effects, so we used a regression model with beta distribution for modeling neural (phase consistency is a unit vector of 0-1 values) and attention data (proportion of correct responses takes 0-1 values). For the attention task, the dependent variable was proportion of correct responses. The categorical variables representing participants’ L1 background (English, Mandarin), musicianship (non-musicians, musicians), domain (speech, tones), and attended dimension (duration, pitch) were coded with a scaled sum contrast with the first variable level coded as -0.5 and the second as 0.5. For cortical neural data, the dependent variable was the mean ITPC across the selected frontocentral channels. For the subcortical data, the dependent variables were the mean ITPC across frequencies of 200–250 Hz or power across frequencies of 100–500 Hz. The categorical variables representing participants’ L1 background, musical training, domain, and for cortical data also acoustic dimension were coded with a scaled sum contrast (-0.5 and 0.5; see above). Across models, participants’ unique IDs were included as a random intercept. As with the categorization data, inclusion of random slopes for domain and dimension resulted in overfitting, so simpler models were used for interpretation (Barr et al., 2013).

Processed data and analysis scripts can be found at:https://osf.io/ajgrn/.

## 3 Results

### 3.1 Effects of L1 experience and music training on dimension-selective attention

**Fig. 2.**
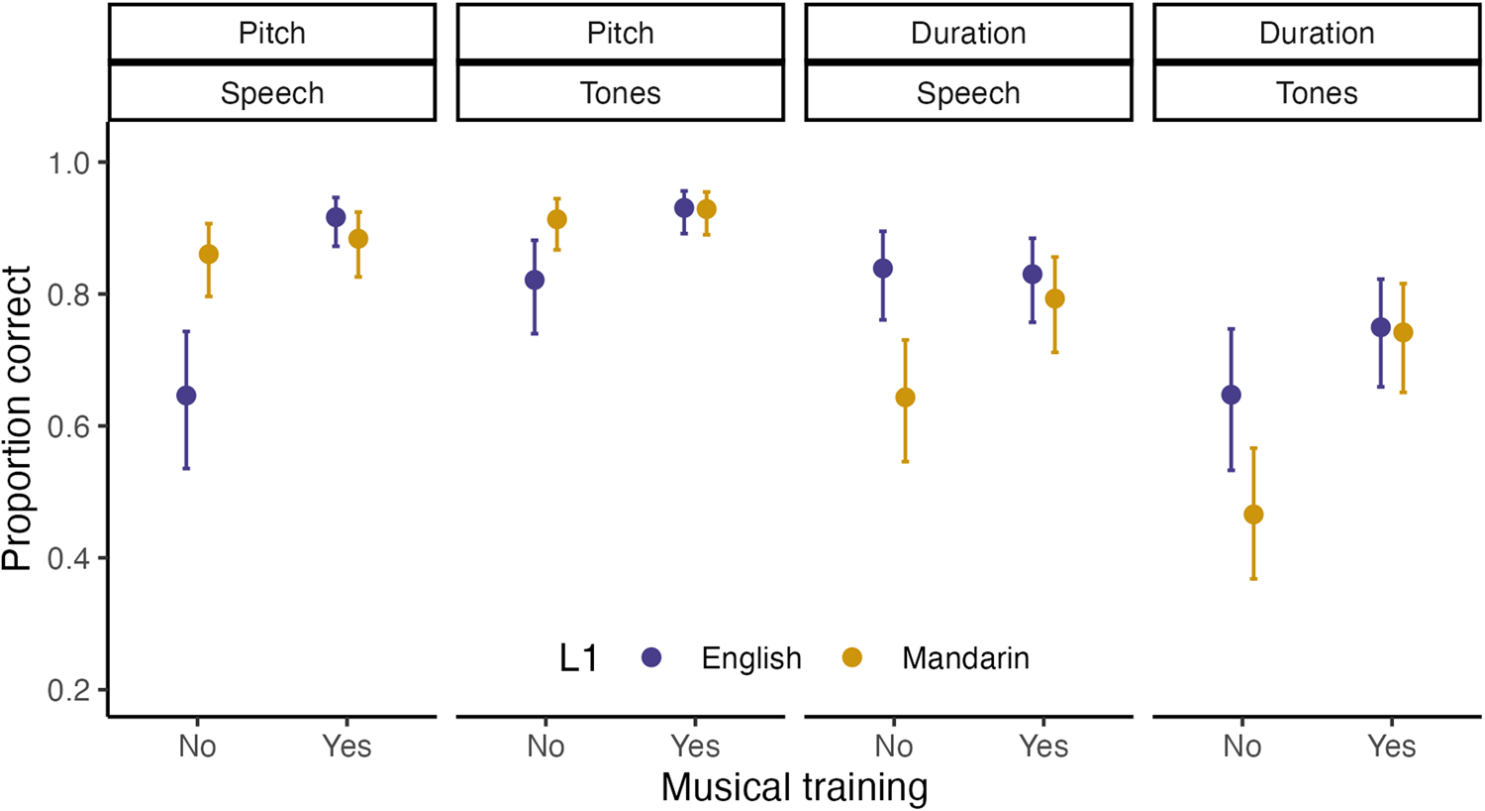
Proportion of correct responses on the dimension-selective attention task for Mandarin and English musicians and non-musicians. Responses were averaged across participants; error bars depict the 95% CI. When attending to pitch and duration, Mandarin-speaking musicians performed equally well compared to native-English-speaking musicians. However, for non-musicians, native Mandarin speakers performed worse than English speakers on attention to duration but better on attention to pitch.

Although participants performed slightly better overall on the dimension-selective attention to pitch relative to duration (Table 1,Fig. 2; main effect of dimension; β = -1.012, p < .001), Mandarin speakers' performance on attending to pitch relative to duration was higher than in native English speakers (interaction between L1 and attended dimension; β = .935, p < .001), indicating a link between tone language experience and enhanced selective attention to pitch. Across all participants, performance was better for pitch relative to duration in tones, but was better for duration relative to pitch in speech (interaction between domain and attended dimension; β = 1.186, p < .001). Musical training also modulated performance across conditions, with better attention performance by musicians compared to non-musicians (main effect of musicianship; β = -.707, p < .001).

**Table 1:** Summary of effects in mixed-effects regression model for dimension-selective attention task.

Predictor	Estimate	SE	z	p
Intercept	1.480	.090	16.491	**<.001**
L1 (English)	.055	.164	.333	.739
Music (non-musicians)	-.707	.164	-4.303	**<.001**
Domain (speech)	.043	.0924	.473	.636
Dimension (duration)	-1.012	.101	-10.019	**<.001**
L1 x music	-.230	.327	-.702	.482
L1 x domain	.117	.185	.635	.525
Music x domain	.068	.185	.367	.714
L1 x dimension	.935	.192	4.859	**<.001**
Music x dimension	.229	.189	1.212	.226
Domain x dimension	1.186	.189	6.287	**<.001**
L1 x music x domain	-.306	.370	-.828	.407
L1 x music x dimension	1.979	.384	5.146	**<.001**
L1 x domain x dimension	.283	.370	.765	.444
Music x domain x dimension	.856	.371	2.309	**.021**
L1 x music x domain x dimension	.841	.741	1.135	.256

Bold values denote statistical significance at the p < 0.05 level.

We found a significant three-way interaction between L1, musical training, and attended dimension (β = 1.979, p < .001). To interpret this interaction, we ran four separate regressions examining the influence of language background on attention performance, examining attention to pitch and duration in musicians and non-musicians. When attending to either pitch or duration, Mandarin-speaking musicians performed equally well compared to native-English-speaking musicians (pitch, β = .16, p = .39; duration, β = .17, p = .63; seeTable S1). However, native-English speaking non-musicians struggled to attend to pitch, while Mandarin-speaking non-musicians performed better (β = -1.00, p < .001). On the other hand, Mandarin-speaking non-musicians struggled to attend to duration, while English-speaking non-musicians performed better (β = .83, p = .004; seeTable S2).

### 3.2 Effects of L1 experience and music training on cue weighting strategies

**Fig. 3.**
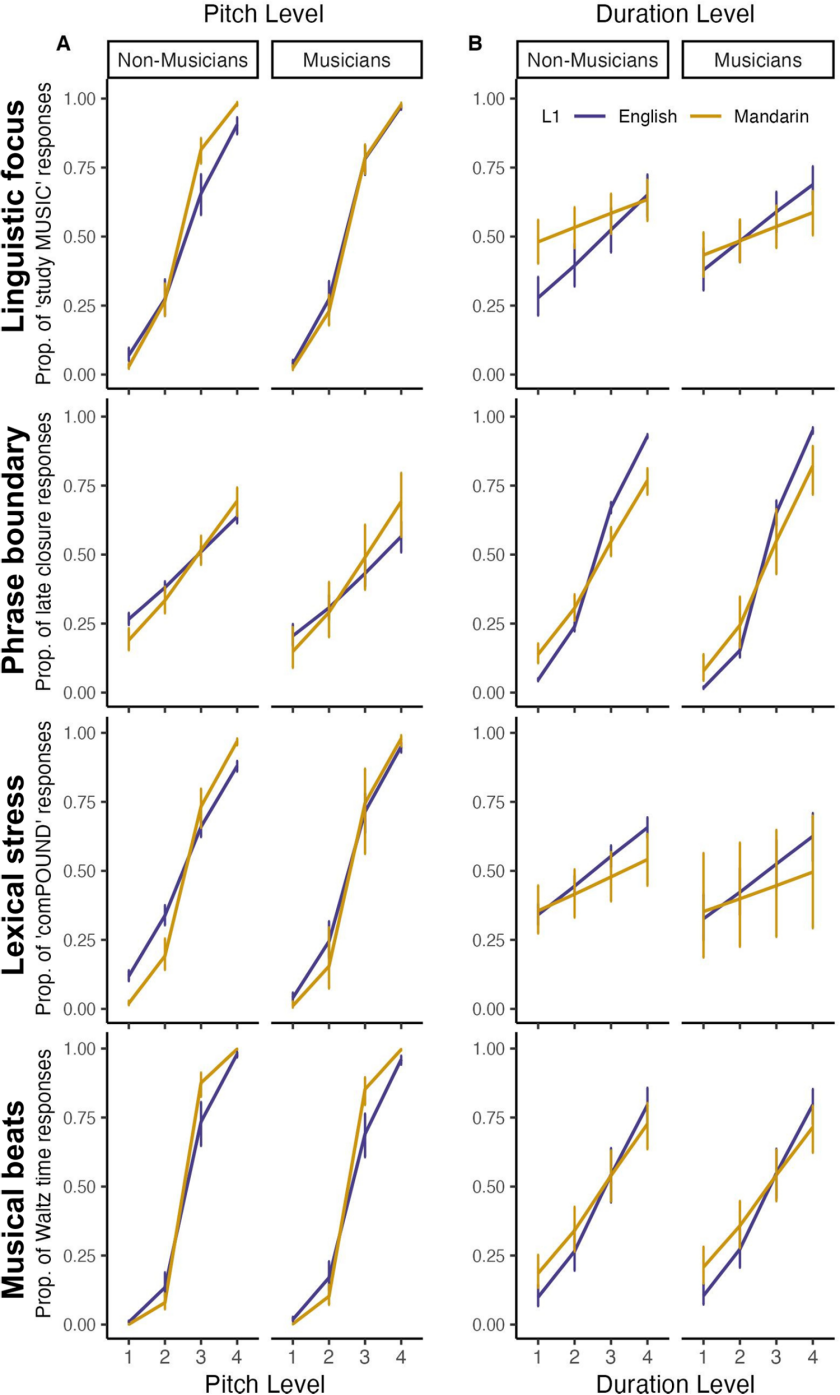
Cue weighting patterns in speech and musical beats categorization tasks. The lines represent the proportion of categorization responses across groups, with error bars depicting 95% CI. Participants’ performance is plotted as a function of pitch level (A) and duration level (B) for Mandarin and English musicians and non-musicians to visualize the differences between the groups in pitch and duration use during categorization. Mandarin speakers relied more on pitch and less on duration than native English speakers across all four categorization tasks.

Across all four categorization tasks, participants were influenced by both acoustic features (Table S3,Fig. 3), confirming that pitch and duration conveyed information about each category (pitch, linguistic focus β = 4.97, p < .001; phrase boundary β = 1.49, p < .001; lexical stress β = 4.81, p < .001; musical beats β = 7.60, p < .001; duration, linguistic focus β = .77, p < .001; phrase boundary β = 3.66, p < .001; lexical stress β = .73, p < .001; musical beats β = 2.20, p < .001). These results indicate that, collapsing across participant groups, pitch was the primary dimension for linguistic focus, lexical stress, and musical beat categorization, while duration was the primary dimension for phrase boundary categorization.

Mandarin speakers relied*more on pitch*than native English speakers across all four categorization tasks, including linguistic focus (interaction between L1 and pitch; β = -1.23, p < .001), phrase boundary (β = -.61, p < .001), lexical stress (β = -2.06, p < .001), and musical beats (β = -3.41, p < .001). Moreover, Mandarin speakers relied*less on duration*across all four categorization tasks: linguistic focus (interaction between L1 and duration; β = .60, p < .001), phrase boundary (β = 2.08, p < .001), lexical stress (β = .45, p < .001), and musical beats (β = .88, p < .001).

A more complex pattern of differences in cue use across tasks was found when comparing musicians and non-musicians. Musicians relied*more on pitch*when categorizing linguistic focus (interaction between musicianship and pitch; β = -1.74, p < .001) and lexical stress (β = -1.17, p < .001), but relied less on pitch when categorizing musical beats (β = 1.08, p < .001). Moreover, musicians relied more on duration when categorizing phrase boundary (β = -.88, p < .001).

Importantly, musicians used duration more as a cue to phrase boundary perception regardless of language background. However, for linguistic focus and lexical stress categorization, three-way interactions between L1, musicianship, and pitch level (focus, β = -1.38, p < .001; stress, β = -.86, p < .001) indicated that Mandarin-speaking and native-English-speaking musicians and non-musicians differed in their pitch reliance. To follow up on these interactions, we ran two separate regression models for Mandarin speakers and native English speakers for each categorization task (see Table S4). These post-hoc analysis revealed that for linguistic focus there was no significant difference between Mandarin-speaking musicians and non-musicians in their pitch use (p > .05), but native-English-speaking musicians relied on pitch more than non-musicians (β = 1.45, p < .001). However, musicians in both language groups relied more on pitch for lexical stress compared to non-musicians (Mandarin speakers (β = -.72, p = .001), native English speakers (β = -1.60, p < .001)).

### 3.3 Effects of L1 experience and music training on dimensional salience measured by EEG-based cortical tracking

**Fig. 4.**
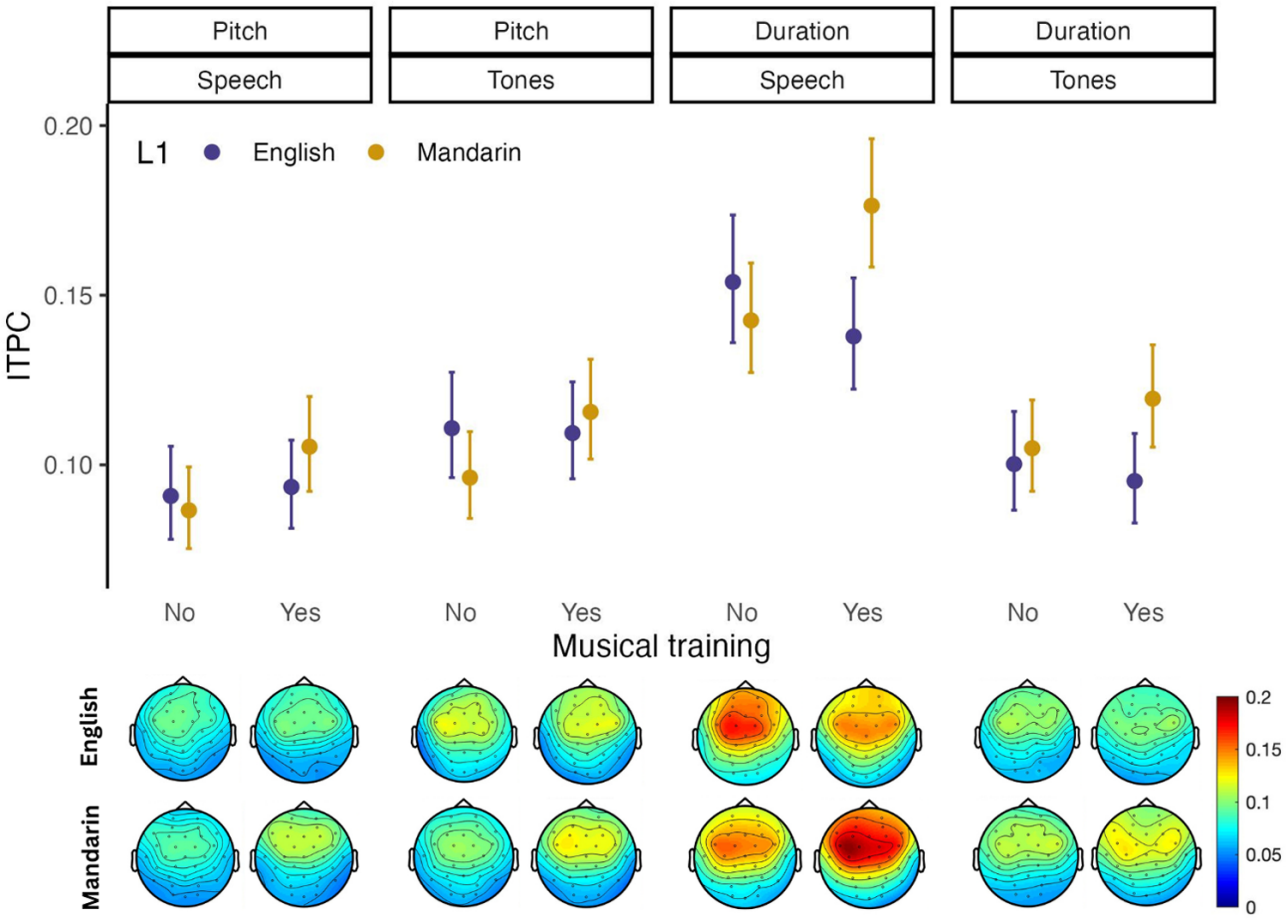
Average ITPC of Mandarin and English musicians and non-musicians at the frequencies corresponding to variations in duration and pitch for each domain (speech, tones) across the frontocentral channels selected for analysis. For individual plots of representative participants seeFigures S1 and S2.

Contra our predictions, there was no effect of language background or musical training on relative cortical tracking of pitch versus duration (Table 2,Fig. 4; no significant interaction of L1 and dimension or musicianship and dimension). However, overall cortical tracking was modulated by a combination of linguistic and musical background, as shown by a significant two-way interaction between L1 and musical training (β = .247, p = .021). Post-hoc regression models for each L1 group with musicianship as a predictor revealed that Mandarin-speaking musicians showed more overall phase-locking compared to the Mandarin-speaking non-musicians (β = -.20, p = .004), whereas there was no difference between native-English-speaking musicians and non-musicians (p > .05). Across both language groups, cortical tracking was greater for speech compared to tones stimuli (β = .137, p < .001) and for duration compared to pitch dimensions (β = .258, p < .001). Relative cortical tracking of dimensions varied with domain (β = .683, p < .001), with greater tracking of pitch for tones compared to speech and greater tracking of duration for speech compared to tones.

**Table 2:** Summary of effects in mixed-effects regression models for ITPC.

Predictor	Estimate	SE	z	p
Intercept	-2.062	.027	-76.30	**<.001**
L1 (English)	-.062	.054	-1.15	.250
Music (non-musicians)	-.082	.054	-1.53	.127
Domain (speech)	.137	.030	4.51	**<.001**
Dimension (duration)	.258	.031	8.43	**<.001**
L1 x music	.247	.107	2.31	**.021**
L1 x domain	-.018	.061	-.130	.766
Music x domain	-.023	.061	-.38	.704
L1 x dimension	-.130	.061	-2.13	**.033**
Music x dimension	.056	.061	.91	.361
Domain x dimension	.683	.061	9.55	**<.001**
L1 x music x domain	.072	.122	.59	.555
L1 x music x dimension	.090	.122	.74	.461
L1 x domain x dimension	.139	.122	1.14	.255
Music x domain x dimension	.012	.122	.09	.924
L1 x music x domain x dimension	.213	.244	.87	.383

Bold values denote statistical significance at the p < 0.05 level.

### 3.4 Effects of L1 experience and music training on neural pitch encoding, as indexed by the frequency following response (FFR)

**Fig. 5.**
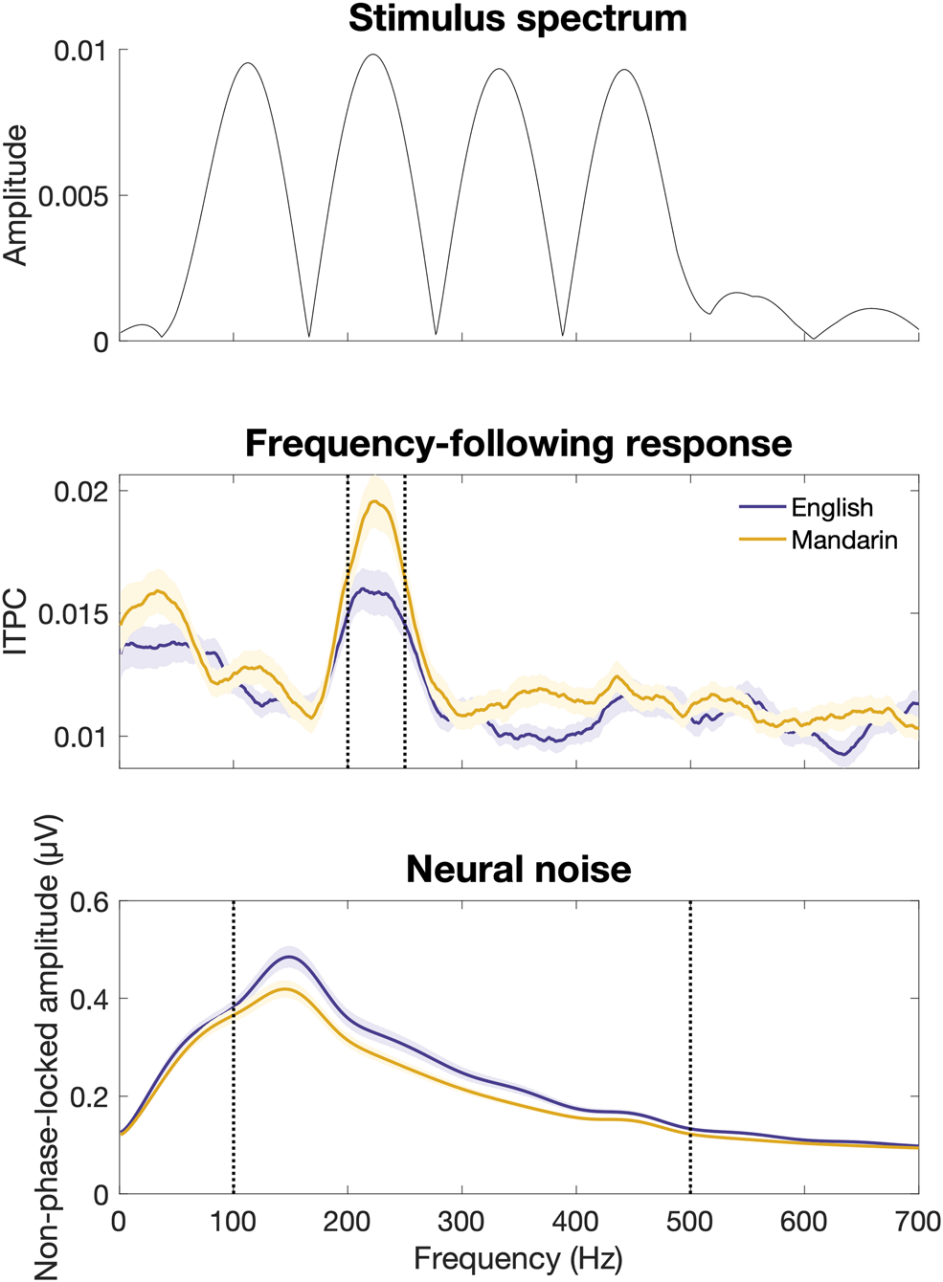
Average ITPC (middle) and non-phase-locked amplitude (bottom) across frequencies for all stimuli collapsed across domains (speech, tones). Dotted lines represent frequency ranges used to compute average ITPC (200*–*250 Hz) and non-phase-locked amplitude (100*–*500 Hz) and frequency of the peak response. For individual plots of representative participants seeFigure S3. For spectrum of the averaged FFR seeFigure S4. The top panel represents the average spectrogram of the lower-pitch speech and tone stimuli included in the analyses, computed with a window size equivalent to that used in the neural analyses.

Mandarin speakers showed more robust early auditory encoding of pitch (FFR ITPC, β = -.158, p = .017) and decreased high-frequency neural noise (FFR Amplitude, β = .034, p = .016) compared to native English speakers (Table 3,Fig. 5). There was no effect of musicianship on either the robustness of auditory encoding or neural noise (p > .05). Across groups, there was a main effect of domain on early auditory encoding of pitch, reflecting greater ITPC to the speech stimulus than the non-speech stimulus (β = .212, p < .001).

Additional analyses including gender and age as covariates (Tables S5–S8) and correlations between behavioural and neural measures (Tables S9–S11) are available in the Supplementary Material. These analyses are not discussed in the main text, as they do not alter the interpretation of the core findings.

**Table 3:** Summary of effects in mixed-effects regression model for ITPC and power.

Predictor	FFR ITPC model				FFR amplitude model			
	Estimate	SE	z	p	Estimate	SE	z	p
Intercept	-4.079	.035	-115.21	**<.001**	.260	.007	36.93	**<.001**
L1 (English)	-.158	.066	-2.38	**.017**	.034	.014	2.40	**.016**
Music (non-musicians)	.026	.066	.040	.689	<.001	.014	-.01	.992
Domain (speech)	.212	.057	3.73	**<.001**	.008	.005	1.61	.106
L1 x music	.030	.132	.22	.822	-.038	.028	-1.35	.176
L1 x domain	.152	.114	1.33	.182	.003	.010	.35	.723
Music x domain	-.017	.114	-.15	.882	-.016	.010	-1.61	.108
L1 x music x domain	.098	.228	.43	.668	-.038	.019	-1.96	.0501

Bold values denote statistical significance at the p < 0.05 level.

## 4 Discussion

### 4.1 Effects of language background

We show that Mandarin speakers up-weight pitch information across speech categorization tasks, including perception of lexical stress, linguistic focus, and phrase boundaries, relative to native English speakers. These results are in line with previous work, which has found greater reliance on pitch among tone language speakers during perception of several English suprasegmental features, including stress (Nguyễn et al., 2008;Wang, 2008;Yu and Andruski, 2010;Y. Zhang et al., 2008;Y. Zhang & Francis, 2010; but seeChrabaszcz et al., 2014) and phrase boundaries (Jasmin, Sun, & Tierney, 2021;Petrova et al., 2023;Zhang, 2012). Moreover, we find that this up-weighting of pitch among Mandarin speakers is not limited to speech perception, extending to perception of musical beats (replicatingJasmin, Sun, & Tierney, 2021andPetrova et al., 2023). This suggests that a domain-general mechanism may underlie shifts in perceptual strategies due to first language experience. One possible candidate is an increase in the salience, or tendency to capture attention, of dimensions which are highly relevant to speech categorization in an individual’s first language (Francis & Nusbaum, 2002;Gordon et al., 1993;Holt et al., 2018).

These attention-to-dimension models of L1 influence on L2 speech perception are supported by our finding that the Mandarin speakers, compared to native English speakers, were better able to selectively attend to pitch but performed*worse*on attending to duration. It was previously reported that L1 Mandarin speakers are better able to attend to pitch in speech but have difficulty ignoring pitch and attending to amplitude (Jasmin, Sun, & Tierney, 2021;Petrova et al., 2023). However, importantly, here we show for the first time that this enhanced attention to pitch and difficulty attending to other dimensions also extends to non-verbal stimuli. This confirms that language experience can have domain-general effects on the ability to attend to sound dimensions. That attention to pitch is enhanced but attention to duration is attenuated in Mandarin speakers could explain recent findings that melodic discrimination is superior (Swaminathan et al., 2018;Zhang, Xie, et al., 2020) but rhythmic discrimination is inferior in tonal compared to non-tonal language speakers (Zhang, Xie, et al., 2020). Interestingly, effects of language experience were found only in non-musicians, while the Mandarin-L1 and English-L1 musicians showed similar performance on the attention to pitch and attention to duration tests. This suggests that musical training can boost the ability to attend to dimensions that would otherwise be difficult to focus on, due to one’s language background.

Despite our finding that Mandarin speakers showed enhanced attention to and preferential use of pitch across*behavioral*tasks, there was no effect of language background on cortical tracking of acoustic dimensions. One possible explanation of these results is that this cortical tracking measure either does not reflect dimensional salience or is insufficiently sensitive to pick up relatively subtle individual differences in dimensional salience patterns. However, we have previously shown that this measure can detect task-driven selective attention to acoustic dimensions and is sensitive to F0 step size (Symons et al., 2021). It is possible that in the absence of appropriate context, the salience of auditory dimensions might not be sufficient to capture a listener’s attention. In other words, attentional capture may be driven by a combination of dimensional biases and context. One way to test this possibility would be to conduct a follow-up study using an experimental paradigm similar to the one reported in this study, but with linguistically meaningful stimuli (e.g., one syllable words). Another possibility is that tone language speakers only experience increased pitch salience in the context of ecologically valid continuous speech. This possibility could be tested by comparing tracking of pitch versus amplitude envelope in naturalistic speech between tone language and non-tone-language speakers using the multivariate temporal response function technique (Crosse et al., 2016,^2021^). Yet another possible explanation of these results is that although tone language speakers benefit from an enhanced ability to direct endogenous attention to pitch when it is task-relevant, they do not experience increased involuntary exogenous capture of attention by pitch.

Although we found no effect of language background on cortical tracking of pitch, we did find that the frequency-following response to stimulus pitch was enhanced in Mandarin speakers. Specifically, we found that Mandarin speakers had enhanced inter-trial phase locking at the frequency of the first harmonic of the F0, as well as decreased non-phase-locked amplitude (“neural noise”) within the frequency range of the FFR (100-500 Hz). One possible mechanism underlying the Mandarin-speaker advantage for FFR encoding, therefore, is this decreased neural noise. These results are in line with previous work linking tone language experience to enhanced FFR pitch tracking (Krishnan et al., 2005,^2010^). Given that the FFR primarily reflects subcortical generators (Bidelman, 2018), with only a modest contribution from cortical sources (Coffey et al., 2017), this suggests that language experience preferentially affects the early stages of auditory processing. An alternate explanation of up-weighting of pitch during perceptual categorization, therefore, is that tone language experience sharpens the precision of early auditory encoding of pitch. This enhanced pitch reliability could result in enhanced use of pitch relative to other dimensions, following models where cue use reflects the relative reliability of acoustic dimensions in signaling speech categories (Toscano & McMurray, 2010). This explanation is supported by prior findings that pitch discrimination thresholds are lower in tone language speakers (Bidelman et al., 2013;Giuliano et al., 2011;Hutka et al., 2015;Pfordresher & Brown, 2009;Zheng & Samuel, 2018; but seeBent et al., 2006;Burns & Sampat, 1980;Peretz et al., 2011;Stagray & Downs, 1993), as well as findings that individuals with poor pitch perception abilities down-weight pitch as a cue during suprasegmental speech categorization (Jasmin et al., 2020).

Despite the prevalent preference for pitch among Mandarin speakers, we also observed a high degree of individual variability in their responses. Some individuals had an extreme pitch bias during phrase boundary categorization or relied less on pitch while categorizing other stimuli where it was the most useful cue (i.e., lexical stress and linguistic focus). These differences indicate that alongside language and musical expertise, individual factors might contribute to shaping perceptual strategies (e.g., attentional control and working memory,Ou et al., 2015; attentional switching,Ou & Law, 2017), which could be investigated in future work.

### 4.2 Effects of music experience

Musical training was linked to sharper tuning to primary dimensions in the L1 English speakers' behavior, consistent with results presented bySymons and Tierney (2023). Specifically, we found that English-speaking musicians showed stronger reliance on pitch for focus and stress categorization compared to English-speaking non-musicians, but stronger reliance on duration for phrase perception. This suggests that, despite their extensive experience with pitch, English-speaking musicians do not broadly up-weight pitch during English speech perception, but instead more highly weight whatever dimension conveys a useful cue for perception of a particular speech category. Importantly, for phrase perception, there was no interaction between musicianship and language background, with both native English and native Mandarin speakers showing an increase in weighting of duration. This suggests that musical training can help listeners make greater use of the primary cue for a particular speech categorization task, even in cases where this goes against the default strategy of a listener’s L1. On the other hand, musicianship interacted with language background for focus and stress perception, with Mandarin-speaking musicians up-weighting pitch less relative to non-musicians; this likely reflects a ceiling effect, given that Mandarin-speaking non-musicians almost entirely use pitch for these categorization tasks.

This finding adds to a large body of work showing that musical training leads to improvements in various aspects of auditory processing (Tervaniemi, 2009). However, the extent and nature of these enhancements might be specific to the type of auditory exposure (Micheyl et al., 2006;Zaltz et al., 2017). For example, research showed that both musicians and audio engineers have generally lower pitch sensitivity thresholds than those without training (Caprini et al., 2024). However, the patterns of advantage are modulated by the specifics of training—while musicians and engineers performed similarly in pitch discrimination tasks, they exhibited differences in sustained selective attention and sound memory tasks (Caprini et al., 2024). In another study, professional violinists and pianists did not differ from each other on auditory psychoacoustic measures, but showed different intonation sensitivity when frequency differences were presented in a musically relevant context of an instrument-specific tuning system (Carey et al., 2015). An interesting avenue for future research could be to investigate whether musical training focused on melodic structure leads to more pitch-biased strategies compared to training concentrated on temporal aspects of music.

We do not replicate prior reports that musical training is linked to an increase in FFR encoding of pitch (Bidelman et al., 2011a,2011b;Skoe & Kraus, 2012;Wong et al., 2007). We do, however, find greater cortical tracking of both dimensions (pitch and duration) in Mandarin-speaking musicians compared to non-musicians, across both verbal and non-verbal stimuli. It is not clear why this pattern was found for the Mandarin speakers but not for the native English speakers. One possibility is that this reflects cultural differences in the way musical training is carried out, either in its intensity or in the aspects of music perception and performance on which the training focuses. Supporting this possibility, a recent comparative study between Chinese traditional and Western music emphasized several important differences (W. Hao, 2023); for example, Chinese music places more emphasis on melodic structure, whereas Western music focuses more on rhythm and harmony. Future work could investigate how cultural differences in music training practice modulate the neural effects of music experience.

### 4.3 Relative dimensional salience differs across domains

Symons et al. (2023)observed that dimensional salience and attention differed across verbal and non-verbal domains: L1 English speakers showed enhanced selective attention and increased cortical tracking of pitch for tone stimuli and duration for speech stimuli, suggesting that attention is guided towards the most relevant dimensions for each domain, facilitating the detection and learning of patterns and categories. The data from L1 English speakers analyzed here is identical to that of Experiment 1 fromSymons et al. (2023). However, here we additionally show that this pattern of enhanced duration salience for verbal stimuli and enhanced pitch salience for non-verbal stimuli extends to L1 Mandarin speakers. While speech and music share certain dimensions, including pitch and duration, they are arguably not equally important across domains (Zatorre & Baum, 2012)—speech is more reliant on rapid temporal information and susceptible to distortions in that dimension (Albouy et al., 2020), whereas music perception is dependent on precise pitch information (Kong et al., 2004). Increased tracking of pitch in musical stimuli and duration in speech stimuli may, therefore, reflect the relative usefulness of those dimensions in each domain.

### 4.4 Limitations

Although we show that the effects of L1 and musical background are not limited to speech stimuli, we cannot make strong claims about the effect of L1 background on music processing, given that our beat categorization test used simple stimuli that do not capture the complexity of ecologically valid music. Future studies should properly examine cue weighting in music perception by including more music-like stimuli. For example, contrasts comprising musical cadences or chord transitions that create a sense of full or partial resolution in musical phrases could be more appropriate for approximating cue weighting in melody perception. Cadential segments, similarly to speech, can be described by multiple cues, for example, by slowing down at structural endings and pitch or harmonic movement toward more stable chords (Palmer & Krumhansl, 1987).

Another limitation of our paradigm was that the stimuli were presented in quiet, which is not indicative of common real-life listening conditions. Most of the time, speech perception takes place in noisy environments, so cue weighting might need to be redistributed differently to account for the availability of the acoustic information in such an environment (e.g.,Gordon et al., 1993;Symons et al., 2024). Further research is needed to determine the role of attention in shaping L2 perceptual strategies in more natural conditions.

### 4.5 Conclusions

Overall, these results are consistent with attentional theories of cue weighting, which suggest that listeners redirect their attention toward the most informative or task-relevant dimensions (Francis & Nusbaum, 2002). Specifically, we find that L1 Mandarin speakers who are not musicians have difficulty attending to duration and use this cue less than native speakers during English speech perception, even in cases such as phrase boundary perception where it is the most useful cue. However, Mandarin-speaking musicians demonstrated an enhanced ability to attend to duration and increased use of duration as a cue during phrase perception, suggesting that effects of language background on cue weighting and dimension-selective attention can be modified by experience later in life. We did not observe an effect of L1 background on dimensional salience, as evidenced by the lack of cortical pitch tracking enhancements; language experience, therefore, may affect endogenous control of dimension-selective attention rather than exogenous attentional capture by sound dimensions. However, we did find that language background enhanced the frequency-following response to sound, suggesting that effects of language experience on sound dimension encoding may be specific to the earlier stages of the auditory pathway.

## Supplementary Material

Supplementary Material

## Data Availability

All data and materials associated with this study are available on the Open Science Framework (OSF) at:https://osf.io/ajgrn/.
