# Supplementary material for: Tone language experience enhances dimension-selective attention and subcortical encoding but not cortical entrainment to pitch
Source: Imaging Neurosci (Camb). 2024 Oct 1;2:imag-2-00297. doi: 10.1162/imag_a_00297 (PMC12290533; doi:10.1162/imag_a_00297)
Supplement: Supplementary Material [file imag_a_00297-supp.pdf]

Supplementary information

Post-hoc analyses for dimension-selective attention

Table S1. Summary of effects in mixed-effects regression models for musicians' performance on the dimension-selective attention task, separate models for acoustic dimensions (pitch, duration).

	Pitch Model				Duration Model			
Predictor	***Estimate***	***SE***	***z***	***p***	***Estimate***	***SE***	***z***	***p***
Intercept	2.445	.155	15.804	**<.001**	1.417	.181	7.839	**<.001**
L1 (English)	.162	.190	.856	.392	.166	.344	.482	.630

Table S2. Summary of effects in mixed-effects regression model for non-musicians' performance on the dimension-selective attention to pitch; separate models for acoustic dimensions (pitch, duration).

	Pitch Model				Duration Model			
Predictor	***Estimate***	***SE***	***z***	***p***	***Estimate***	***SE***	***z***	***p***
Intercept	1.594	.159	10.054	**<.001**	.637	.143	4.447	**<.001**
L1 (English)	-.997	.284	-3.514	**<.001**	.825	.286	2.884	**.004**

Effects of L1 experience and music training on cue weighting strategies

Table S3. Summary of effects in mixed effects logistic regression models for speech and musical beats categorization tasks

Task	***Predictor***	***Estimate***	***SE***	***z***	***p***
Linguistic Focus					
	Intercept	.065	.079	.823	.410
	L1 (English)	-.147	.158	-.932	.351
	Music (non-musicians)	-.059	.158	-.372	.710
	Pitch	4.965	.071	70.387	**<.001**
	Duration	.766	.046	16.614	**<.001**
	L1 x Music	-.508	.315	-1.612	.107
	L1 x Pitch	-1.266	.139	-9.119	**<.001**
	Music x Pitch	-.735	.139	-5.299	**<.001**
	L1 x Duration	.602	.092	6.531	**<.001**
	Music x Duration	.110	.092	1.194	.232
	Pitch x Duration	.318	.128	2.482	**.013**
	L1 x Music x Pitch	-1.384	.279	-4.968	**<.001**
	L1 x Music x Duration	.211	.184	1.148	.251
	L1 x Pitch x Duration	-.321	.256	-1.257	.209
	Music x Pitch x Duration	-.396	.256	-1.549	.121
	L1 x Music x Pitch x Duration	-.288	.510	-.566	.572
Phrase Boundary					
	Intercept	-.387	.046	-8.398	**<.001**
	L1 (English)	.006	.092	.069	.945
	Music (non-musicians)	.236	.092	2.559	**.011**
	Pitch	1.493	.045	33.538	**<.001**
	Duration	3.657	.057	64.619	**<.001**
	L1 x Music	.168	.184	.911	.362

	L1 x Pitch	-.605	.089	-6.816	**<.001**
	Music x Pitch	-.121	.089	-1.359	.174
	L1 x Duration	2.084	.112	18.564	**<.001**
	Music x Duration	-.881	.112	-7.857	**<.001**
	Pitch x Duration	-.083	.108	-.766	.443
	L1 x Music x Pitch	.186	.178	1.047	.295
	L1 x Music x Duration	-.330	.224	-1.470	.142
	L1 x Pitch x Duration	-.063	.216	-.292	.711
	Music x Pitch x Duration	.151	.216	.701	.484
	L1 x Music x Pitch x Duration	-.083	.430	-.193	.847
Lexical Stress					
	Intercept	-.160	.081	-1.976	**.048**
	L1 (English)	.209	.161	1.292	.196
	Music (non-musicians)	.100	.161	.617	.538
	Pitch	4.809	.070	68.559	**<.001**
	Duration	.726	.046	15.921	**<.001**
	L1 x Music	.003	.323	.010	.992
	L1 x Pitch	-2.061	.138	-14.890	**<.001**
	Music x Pitch	-1.167	.138	-8.416	**<.001**
	L1 x Duration	.450	.091	4.884	**<.001**
	Music x Duration	.090	.091	.988	.323
	Pitch x Duration	.203	.128	1.587	.113
	L1 x Music x Pitch	-.860	.277	-3.104	**<.001**
	L1 x Music x Duration	-.076	.182	-.418	.676
	L1 x Pitch x Duration	-.635	.256	-2.479	**.013**
	Music x Pitch x Duration	-.280	.256	-1.093	.274
	L1 x Music x Pitch x Duration	.895	.512	1.748	.081
Musical Beats					
	Intercept	-.319	.096	-3.333	**.001**
	L1 (English)	-.178	.191	-.932	.351
	Music (non-musicians)	-.037	.191	-.192	.848
	Pitch	7.602	.128	59.162	**<.001**
	Duration	2.198	.066	33.448	**<.001**
	L1 x Music	.010	.382	.027	.979
	L1 x Pitch	-3.413	.252	-13.535	**<.001**
	Music x Pitch	1.077	.250	4.308	**<.001**
	L1 x Duration	.881	.130	6.749	**<.001**
	Music x Duration	.093	.130	.712	.476
	Pitch x Duration	4.203	.207	20.288	**<.001**
	L1 x Music x Pitch	.034	.500	.069	.945
	L1 x Music x Duration	-.112	.261	-.428	.669
	L1 x Pitch x Duration	-.825	.412	-2.001	**.045**
	Music x Pitch x Duration	2.182	.412	5.301	**<.001**
	L1 x Music x Pitch x Duration	.576	.821	.702	.483

Post-hoc analyses for cue weighting categorization tasks

Table S4. Summary of effects of mixed-effects regression models for linguistic focus and lexical stress categorization tasks; separate models for participant's L1 (Mandarin, English).

	L1 Mandarin Model				***L1 English Model***			
Predictor	***Estimate***	***SE***	***z***	***p***	***Estimate***	***SE***	***z***	***p***
Linguistic Focus								
Intercept	.128	.105	1.220	.223	-.012	.112	-.108	.914
Music (Non-Musicians)	.198	.209	.948	.343	-.275	.225	-1.224	.221
Pitch	5.530	.107	51.742	**<.001**	4.128	.085	48.600	**<.001**
Music x Pitch	-.036	.211	-.169	.866	-1.450	.168	-8.619	**<.001**
Lexical Stress								
Intercept	-.269	.084	-3.179	**.001**	-.050	.136	-.364	.716
Music (Non-Musicians)	.109	.169	.646	.518	.093	.272	.341	.733
Pitch	5.709	.111	51.332	**<.001**	3.659	.078	46.706	**<.001**
Music x Pitch	-.724	.219	-3.306	**<.001**	-1.603	.156	-10.309	**<.001**

Effects of L1 experience and music training on dimensional salience measured by EEG-based cortical tracking

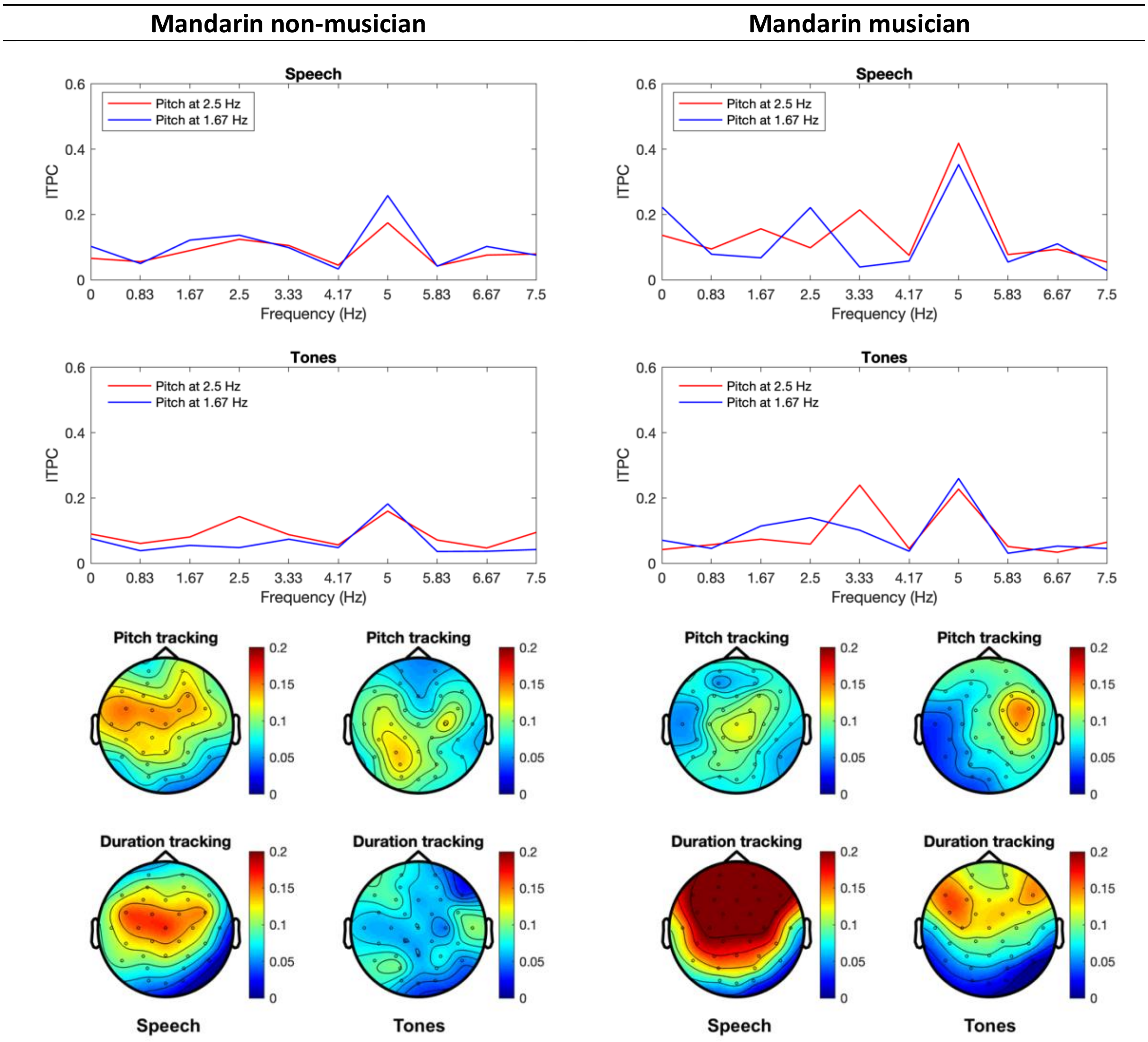

Figure S1. ITPC of representative participants from Mandarin musician and non-musician groups at the frequencies corresponding to variations in duration and pitch for each domain (speech, tones) across the frontocentral channels selected for analysis. Top panels show the ITPC over a range of frequencies with separate lines corresponding to different rates of stimuli presentation. The topographical plots display the distribution of brain responses to pitch and duration for speech (left) and tones (right) stimuli.

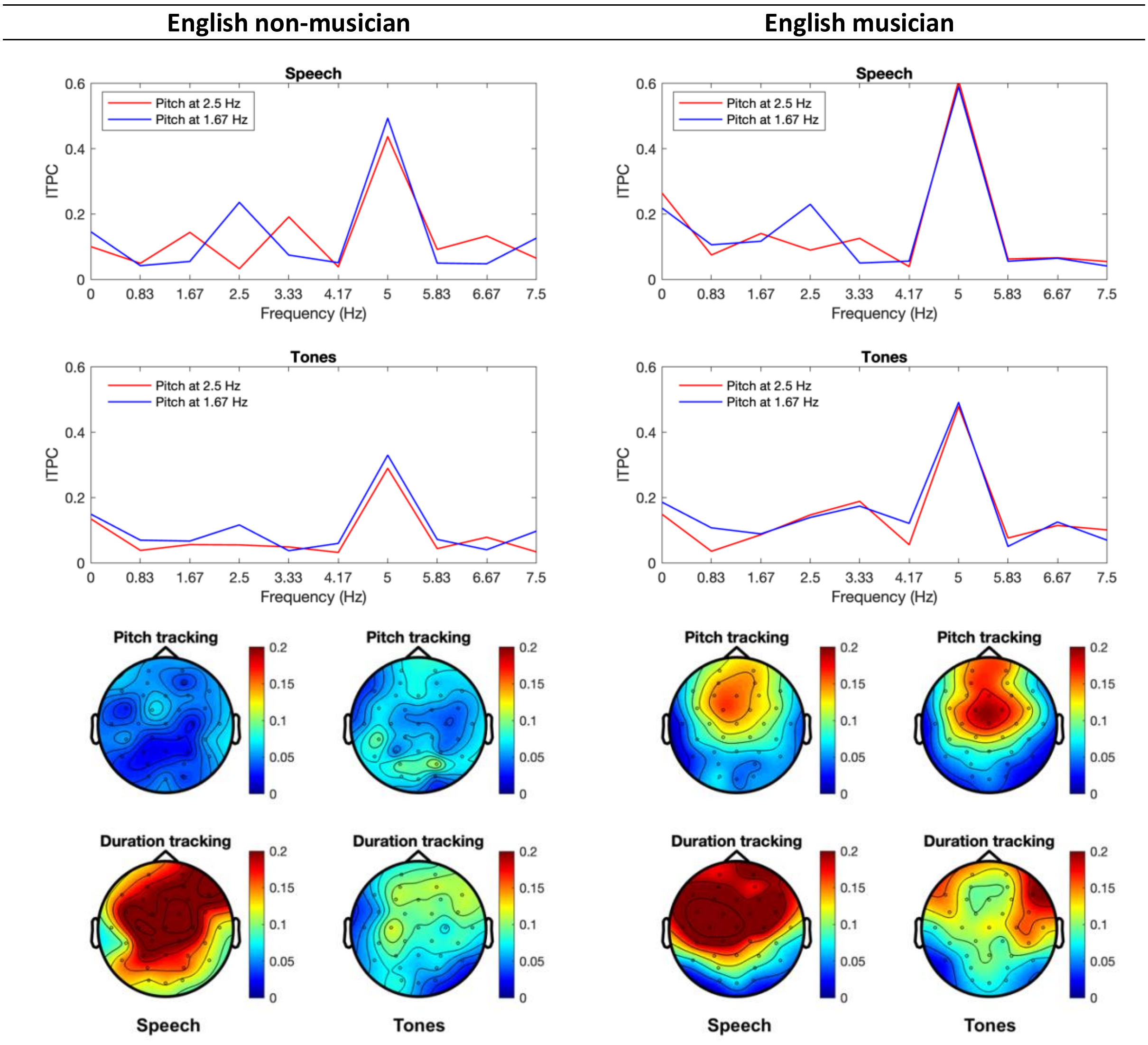

Figure S2. ITPC of representative participants from English musician and non-musician groups at the frequencies corresponding to variations in duration and pitch for each domain (speech, tones) across the frontocentral channels selected for analysis. Top panels show the ITPC over a range of frequencies with separate lines corresponding to different rates of stimuli presentation. The topographical plots display the distribution of brain responses to pitch and duration for speech (left) and tones (right) stimuli.

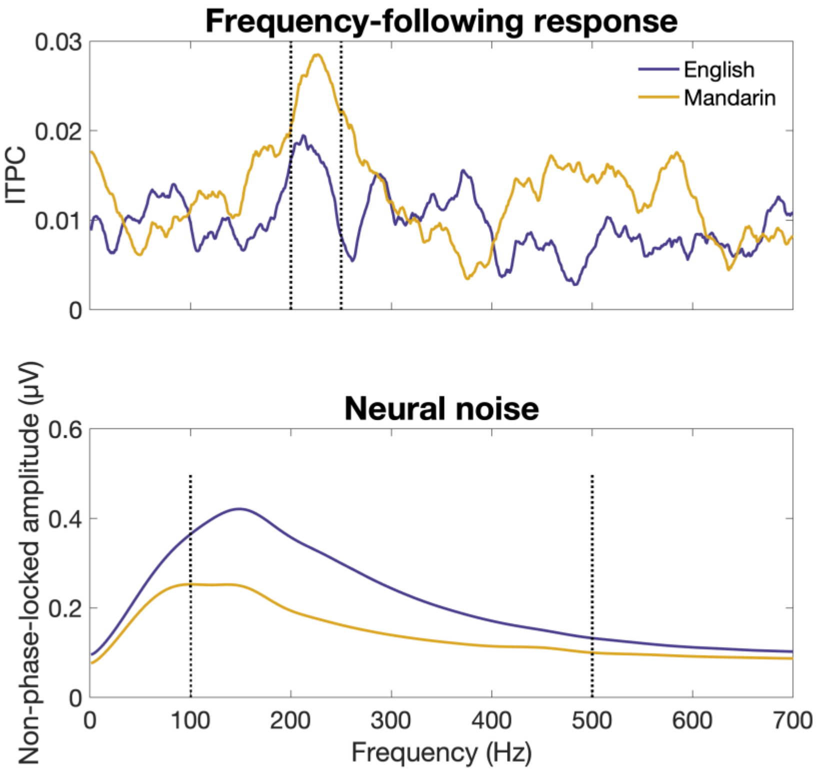

Figure S3. ITPC (top) and non-phase locked amplitude (bottom) across frequencies for all stimuli collapsed across domains (speech, tones) for one representative Mandarin and one English participant. Dotted lines represent frequency ranges used to compute average ITPC (200-250 Hz) and non-phase locked amplitude (100-500 Hz).

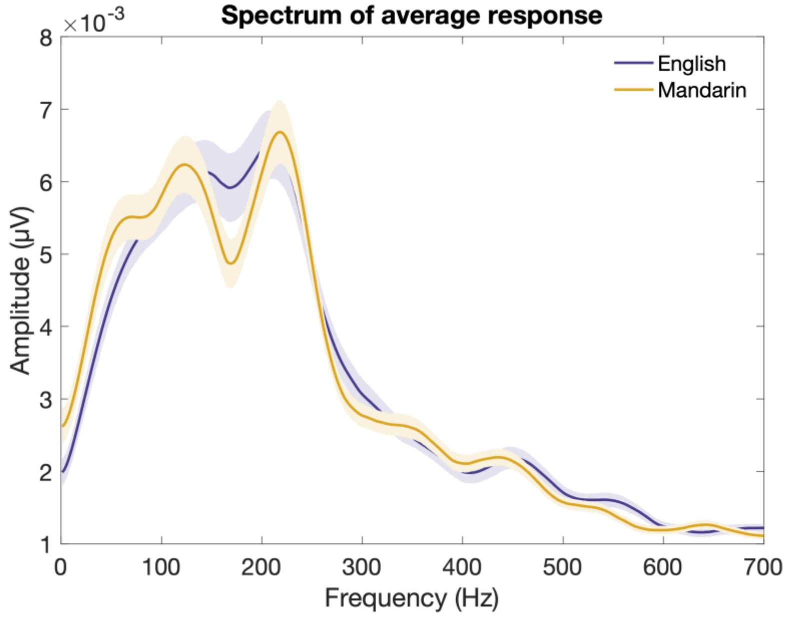

Figure S4. Amplitude spectrum of the average waveform collapsed across domains (speech, tones). Dotted lines represent frequency ranges used to compute average ITPC (200-250 Hz) and non-phase-locked amplitude (100-500 Hz).

Analyses with age and gender as covariates

Table S5. Summary of effects in mixed-effects regression model for dimension-selective attention task with age and gender as covariates.

Predictor	*Estimate*	*SE*	*z*	*p*
Intercept	1.489	.312	4.767	**<.001**
L1 (English)	.050	.169	.295	.768
Music (Non-Musicians)	-.740	.165	-4.476	**<.001**
Domain (Speech)	.043	.092	. 466	.641
Dimension (Duration)	-1.040	.109	-10.046	**<.001**
Age	-.283	.173	-1.638	.101
Gender: Male	-.046	.314	-.147	.883
Gender: Non-conforming	.140	.330	.425	.671
L1 x Music	-.315	.342	-.921	.357
L1 x Domain	.116	.185	.627	.531
Music x Domain	.065	.185	.353	.724
L1 x Dimension	.940	.192	4.890	**<.001**
Music x Dimension	.223	.189	1.182	.237
Domain x Dimension	1.183	.187	6.269	**<.001**
L1 x Music x Domain	-.305	.369	-.826	.409
L1 x Music x Dimension	1.972	.384	5.132	**<.001**
L1 x Domain x Dimension	.287	.370	.744	.438
Music x Domain x Dimension	.846	.371	2.283	**.022**
L1 x Music x Domain x Dimension	.832	.740	1.124	.261

Table S6. Summary of effects in mixed effects logistic regression models for speech and musical beats categorization tasks with age and gender as covariates

Task	*Predictor*	*Estimate*	*SE*	*z*	*p*
Linguistic Focus					
	Intercept	.331	.284	1.165	.244
	L1 (English)	-.247	.160	-1.538	.124
	Music (non-musicians)	-.020	.156	-.130	.896
	Pitch	4.965	.071	70.386	**<.001**
	Duration	.766	.046	16.613	**<.001**
	Age	.216	.163	1.322	.186
	Gender: Male	-.349	.289	-1.210	.227
	Gender: Non-conforming	.042	.304	.138	.890
	L1 x Music	-.243	.324	-.749	.454
	L1 x Pitch	-1.262	.139	-9.091	**<.001**
	Music x Pitch	-.743	.139	-5.348	**<.001**
	L1 x Duration	.602	.092	6.534	**<.001**
	Music x Duration	.108	.092	1.176	.239
	Pitch x Duration	.318	.128	2.483	**.013**
	L1 x Music x Pitch	-1.399	.279	-5.021	**<.001**
	L1 x Music x Duration	.208	.184	1.130	.258
	L1 x Pitch x Duration	-.321	.256	-1.254	.210
	Music x Pitch x Duration	-.396	.256	-1.548	.122
	L1 x Music x Pitch x Duration	-.289	.509	-.565	.572
Phrase Boundary					

Intercept	-.490	.163	-2.999	**.003**
L1 (English)	-.016	.096	-.166	.868
Music (non-musicians)	.251	.093	2.694	**.007**
Pitch	1.493	.045	33.538	**<.001**
Duration	3.656	.057	64.619	**<.001**
Age	.053	.097	.550	.582
Gender: Male	.096	.166	.581	.561
Gender: Non-conforming	.143	.175	.817	.414
L1 x Music	.200	.194	1.035	.301
L1 x Pitch	-.606	.089	-6.819	**<.001**
Music x Pitch	-.120	.089	-1.354	.176
L1 x Duration	2.084	.112	18.553	**<.001**
Music x Duration	-.880	.112	-7.845	**<.001**
Pitch x Duration	-.082	.108	-.766	.443
L1 x Music x Pitch	.186	.178	1.046	.296
L1 x Music x Duration	-.329	.224	-1.468	.142
L1 x Pitch x Duration	-.063	.216	-.292	.717
Music x Pitch x Duration	.151	.216	.701	.483
L1 x Music x Pitch x Duration	-.083	.430	-.194	.846
Lexical Stress				
Intercept	.047	.298	.156	.876
L1 (English)	.190	.168	1.130	.259
Music (non-musicians)	.094	.163	.572	.568
Pitch	4.809	.070	68.559	**<.001**
Duration	.726	.046	15.920	**<.001**
Age	-.021	.171	-.124	.901
Gender: Male	-.241	.303	-.795	.428
Gender: Non-conforming	-.094	.319	-.294	.769
L1 x Music	.061	.340	.179	.858
L1 x Pitch	-2.062	.138	-14.891	**<.001**
Music x Pitch	-1.168	.139	-8.420	**<.001**
L1 x Duration	.445	.091	4.883	**<.001**
Music x Duration	.090	.091	.986	.324
Pitch x Duration	.203	.128	1.587	.113
L1 x Music x Pitch	-.863	.277	-3.114	**.002**
L1 x Music x Duration	-.077	.182	-.422	.673
L1 x Pitch x Duration	-.635	.256	-2.479	**.013**
Music x Pitch x Duration	-.280	.256	-1.093	.274
L1 x Music x Pitch x Duration	.895	.513	1.750	.080
Musical Beats				
Intercept	-.057	.350	-.162	.871
L1 (English)	-.146	.199	-.732	.464
Music (non-musicians)	-.051	.194	-.262	.793
Pitch	7.602	.128	59.142	**<.001**
Duration	2.198	.066	33.448	**<.001**
Age	.029	.199	.147	.883
Gender: Male	-.247	.356	-.693	.489
Gender: Non-conforming	-.356	.375	-.950	.969
L1 x Music	.016	.402	.039	.969
L1 x Pitch	-3.414	.252	-13.534	**<.001**
Music x Pitch	1.079	.250	4.317	**<.001**

L1 x Duration	.881	.130	6.749	**<.001**
Music x Duration	.093	.207	.712	.476
Pitch x Duration	4.203	.207	20.287	**<.001**
L1 x Music x Pitch	.027	.497	.055	.956
L1 x Music x Duration	-.113	.260	-.435	.664
L1 x Pitch x Duration	-.825	.412	-2.003	**.045**
Music x Pitch x Duration	2.183	.411	5.310	**<.001**
L1 x Music x Pitch x Duration	.572	.817	.699	.484

Table S7. Summary of effects in mixed effects regression models for ITPC with age and gender as covariates

**Predictor**	**Estimate**	**SE**	**z**	**p**
Intercept	-2.304	.104	-22.061	**<.001**
L1 (English)	-.067	.053	-1.271	.204
Music (Non-musicians)	-.055	.052	-1.076	.282
Domain (Speech)	.137	.030	4.504	**<.001**
Dimension (Duration)	.261	.031	8.516	**<.001**
Age	.141	.054	2.620	**.009**
Gender: Male	.271	.106	2.564	**.010**
Gender: Non-conforming	.158	.110	1.427	.154
L1 x Music	.262	.107	2.444	**.015**
L1 x Domain	-.015	.061	-.245	.806
Music x Domain	-.026	.061	-.425	.671
L1 x Dimension	-.131	.061	.939	**.032**
Music x Dimension	.057	.061	.939	.348
Domain x Dimension	.582	.061	9.544	**<.001**
L1 x Music x Domain	.072	.122	.592	.554
L1 x Music x Dimension	.086	.122	.704	.481
L1 x Domain x Dimension	.139	.122	1.144	.253
Music x Domain x Dimension	.012	.122	.100	.920
L1 x Music x Domain x Dimension	.216	.244	.885	.376

Table S8. Summary of effects in mixed-effects regression model for ITPC and non-phase-locked amplitude with age and gender as covariates.

	FFR ITPC Model				***FFR Amplitude Model***			
**Predictor**	**Estimate**	**SE**	**z**	**p**	**Estimate**	**SE**	**z**	**p**
Intercept	-4.164	.126	-32.97	**<.001**	.262	.026	10.177	**<.001**
L1 (English)	-.129	.068	-1.90	.057	.028	.015	1.913	.056
Music (Non-Musicians)	.011	.066	.17	.867	.001	.014	.093	.926
Domain (Speech)	.214	.057	3.76	**<.001**	.008	.005	1.614	.107
Age	-.113	.070	-1.63	.104	.002	.015	.108	.914
Gender: Male	.100	.127	.79	.430	-.007	.026	-.286	.775
Gender: Non-conform.	.030	.134	.23	.820	.019	.028	.702	.483
L1 x Music	-.057	.137	-.42	.677	-.028	.029	-.959	.338
L1 x Domain	.152	.114	1.34	.180	.003	.010	.354	.723
Music x Domain	-.018	.113	-.16	.874	-.016	.010	-1.607	.108
L1 x Music x Domain	.080	.227	.35	.726	-.038	.019	-1.960	.050

Relationships between neural and behavioural measures

Table S9. Correlations between ITPC and dimension-selective attention to duration across groups. Reported p-values are not corrected for multiple comparisons.

	Domain: Speech		***Domain: Tones***	
	r	***p***	***r***	***p***
Mandarin group (all participants)	.15	.26	-.099	.45
Musicians only	.079	.68	.18	.35
Non-musicians only	.061	.74	-.36	.045
English group (all participants)	-.048	.73	-.017	.90
Musicians only	.11	.58	.10	.60
Non-musicians only	-.20	.33	-.098	.64

Table S10. Correlations between FFR ITPC and dimension-selective attention to pitch across groups. Reported p-values are not corrected for multiple comparisons.

	Domain: Speech		***Domain: Tones***	
	r	***p***	***r***	***p***
Mandarin group (all participants)	-.16	.22	.05	.7
Musicians only	-.098	.61	.15	.44
Non-musicians only	-.2	.27	-.016	.93
English group (all participants)	.15	.28	.058	.68
Musicians only	-.14	.47	-.26	.18
Non-musicians only	.45	.025	.25	.23

Table S11. Correlations between FFR non-phase-locked amplitude and dimension-selective attention to pitch across groups. Reported p-values are not corrected for multiple comparisons.

	Domain: Speech		***Domain: Tones***	
	r	***p***	***r***	***p***
Mandarin group (all participants)	.18	.18	-.097	.46
Musicians only	.061	.75	-.004	.98
Non-musicians only	.29	.11	-.19	.30
English group (all participants)	.046	.74	-.15	.27
Musicians only	.088	.65	-.27	.15
Non-musicians only	-.21	.32	-.16	.45
